# Reply to: Inconsistent kinetic isotope effect in ammonia charge exchange reaction measured in a Coulomb crystal and in a selected-ion flow tube

**DOI:** 10.1038/s41467-022-30567-2

**Published:** 2022-06-09

**Authors:** L. S. Petralia, A. Tsikritea, J. Loreau, T. P. Softley, B. R. Heazlewood

**Affiliations:** 1 Department of Chemistry, University of Oxford, Physical and Theoretical Chemistry Laboratory, South Parks Road, Oxford, OX1 3QZ UK; 2 Department of Physics, University of Liverpool, The Oliver Lodge, Liverpool, L69 7ZE UK; 3 KU Leuven, Department of Chemistry, Celestijnenlaan 200 F, B-3001 Leuven, Belgium; 4 School of Chemistry, University of Birmingham, Edgbaston, B15 2TT UK

**Keywords:** Reaction kinetics and dynamics, Chemical physics

**replying to** Shaun G. Ard et al. *Nature Communications* 10.1038/s41467-022-30566-3 (2022)

In our 2020 Nature Communications paper^1^, we reported a strong inverse kinetic isotope effect in the charge transfer reactions of NH_3_ and ND_3_ with Xe^+^ (^2^P_3/2_) ions. We subsequently measured progressively smaller inverse isotope effects for the charge transfer reactions of ammonia with Kr^+^ and Ar^+^ ions, following an expected periodic trend^2^. In all systems, experimental measurements have been complemented with detailed theory work and we have proposed a credible explanation for the experimental observations.

In their Matters Arising submission, Ard et al. detail new measurements they have undertaken on the Xe^+^ + NH_3_ and ND_3_ reaction systems^3^. They monitor the decay of Xe^+^ ions using a variable ion source and temperature-adjustable selected-ion flow tube (VISTA-SIFT) apparatus, reporting rate coefficients over a temperature range spanning 175–600 K. Both the VISTA-SIFT measurements and those reported in our paper feature fairly large uncertainty ranges. Uncertainties of ±30% absolute and ±20% relative are stated in the work of Ard et al.;^3^ uncertainties due to systematic errors are estimated to be up to a factor-of-two in our study, alongside smaller relative uncertainties associated with each rate coefficient^1^. Accurately establishing the magnitude of systematic errors is challenging, and we were therefore conservative in our treatment of these sources of uncertainty. Calculating the relative uncertainty in the rate coefficients from the standard error (as set out in reference 2) yields slightly higher uncertainties than reported in our original analysis (where we use the propagation of errors method): 3.6 ± 0.2 × 10^–10^ cm^3^ s^–1^ for NH_3_ and 1.2 ± 0.5 × 10^–9^ cm^3^ s^–1^ for ND_3_. The rate coefficients—and the resulting kinetic isotope effect—are unchanged, but the uncertainty range associated with the isotope effect increases from 0.3 ± 0.05 to 0.3 ± 0.1 with the updated treatment of the relative errors. This compares to the kinetic isotope effect of approximately 0.8 ± 0.2 reported by Ard et al. in figure 2 (at a comparable collision energy)^3^. There are a number of key differences between our experimental methods which potentially inhibit a robust comparison between the reported rate coefficients carried out using VISTA-SIFT^3^ and those reported in our work^1^. It is difficult to directly compare measurements from the two studies as the experiments monitor different things and have been conducted under different conditions.

Ard et al. report the consumption of Xe^+^ ions, adopting the same approach as was taken in the previous SIFT measurement of the Xe^+^ + NH_3_ reaction at 300 K^4^. Ard et al. report that measurements of product formation from unquenched data [i.e., with Xe^+^ present in both the (^2^P_3/2_) and (^2^P_1/2_) states] are consistent with the rate coefficients obtained from monitoring Xe^+^(^2^P_3/2_) consumption. In our experimental set-up, we eject the ions into a mass spectrometer and hence directly confirm the identities of any product ions. No cross contamination could have occurred in our experiments, as this would have been seen in both the residual gas analyser background scans and in the time-of-flight traces. We can account for all species present in the reaction chamber. There are no buffer gases present in our experiment and measurements are carried out under ultra-high vacuum conditions (approximately 1 × 10^–9^ mbar). Our detection sensitivity is such that we would see alternative products formed from collisions with background gases, or from competing reaction channels, in the mass spectra^5^. In the few cases where background reactions do occur in our experiments, these are explicitly accounted for and included in our analysis^1,2^. We observe no competing reaction channels under our experimental conditions. However, we note that an earlier ion cyclotron resonance (ICR) study of the Xe^+^ + NH_3_ reaction discussed the possible contribution of a hydrogen abstraction channel at 300 K, yielding XeH^+^ products^6^.

In our set-up, we can literally ‘see’ the reaction taking place in real time, over the course of a few minutes, through the accumulation of a dark core in the Coulomb crystal images. We can directly observe that ND_3_ reacts noticeably faster than NH_3_, and we are confident that this is a real effect. As stated in the previous paragraph, our measurements are carried out under ultra-high vacuum conditions. The reactants are not in thermal equilibrium; the ionic reactants are effectively stationary and the ammonia reactants are thermal. As Ard et al. note, the measurements performed using the VISTA-SIFT apparatus are carried out under thermal equilibrium and at approximately nine orders of magnitude higher pressures. Calculations reported by Ard et al. suggest that the reaction complex is unlikely to undergo collisions with the helium buffer gas during the calculated lifetime under their conditions^3^. As noted in our initial manuscript, recent studies have uncovered evidence that collision complexes can be long-lived in a number of reaction systems—with these effects not always predicted by statistical methods. It is also unclear whether the presence of He buffer gas (or other gaseous species) in the flow tube might influence the rate of consumption of Xe^+^ ions in other ways.

Only elastic collisions can occur between co-trapped ions in a Coulomb crystal. There are no known collective effects that might influence the short-range dynamics of the reacting ions. For example, there is no sympathetic cooling of rotational or vibrational modes of trapped ions as the interaction range is too long (neighbouring ions are separated by 10–20 μm); the reaction complex therefore will not undergo any inelastic collisions with other co-trapped ions. Over the past decade or so, there have been numerous ion–molecule reaction systems studied in Coulomb crystals under comparable conditions to those we employ. In many cases—including in our own most recent study, involving the charge transfer of H_2_O and D_2_O with Kr^+^ ions—experimental rate coefficients have been in excellent agreement with capture theory predictions^7–10^. For other systems, experimental rate coefficients have been found to be lower than predicted by capture theory^1,2,11–13^. Certain features on the underlying potential energy surfaces, such as the absence of an energetically accessible crossing point between the reactant and product surfaces or the presence of submerged barriers, are known to give rise to reactions that are not capture limited; this is seen in our study of Xe^+^ with ammonia.

As we note in our recent work on the topic, further measurements are needed to confirm the mechanism of charge transfer between ammonia isotopologues and rare-gas ions including Xe^+^^1,2^. We plan to undertake further measurements on this system under different experimental conditions to those explored previously—by controlling and varying both the collision energy and the internal energy of the ammonia reactant.

## Data Availability

Supporting data can be obtained from the original manuscript, 10.1038/s41467-019-13976-8, and from the Oxford Research Archive, 10.5287/bodleian:NoRj6KRde.
